# Assessing Competencies Needed to Engage With Digital Health Services: Development of the eHealth Literacy Assessment Toolkit

**DOI:** 10.2196/jmir.8347

**Published:** 2018-05-10

**Authors:** Astrid Karnoe, Dorthe Furstrand, Karl Bang Christensen, Ole Norgaard, Lars Kayser

**Affiliations:** ^1^ Department of Public Health University of Copenhagen Copenhagen Denmark; ^2^ Danish Multiple Sclerosis Society Valby Denmark; ^3^ Danish Cancer Research Center Danish Cancer Society Copenhagen Denmark; ^4^ Scias Copenhagen Denmark; ^5^ Steno Diabetes Center Copenhagen Gentofte Denmark

**Keywords:** health literacy, computer literacy, questionnaires, telemedicine, consumer health informatics

## Abstract

**Background:**

To achieve full potential in user-oriented eHealth projects, we need to ensure a match between the eHealth technology and the user’s eHealth literacy, described as knowledge and skills. However, there is a lack of multifaceted eHealth literacy assessment tools suitable for screening purposes.

**Objective:**

The objective of our study was to develop and validate an eHealth literacy assessment toolkit (eHLA) that assesses individuals’ health literacy and digital literacy using a mix of existing and newly developed scales.

**Methods:**

From 2011 to 2015, scales were continuously tested and developed in an iterative process, which led to 7 tools being included in the validation study. The eHLA validation version consisted of 4 health-related tools (tool 1: “functional health literacy,” tool 2: “health literacy self-assessment,” tool 3: “familiarity with health and health care,” and tool 4: “knowledge of health and disease”) and 3 digitally-related tools (tool 5: “technology familiarity,” tool 6: “technology confidence,” and tool 7: “incentives for engaging with technology”) that were tested in 475 respondents from a general population sample and an outpatient clinic. Statistical analyses examined floor and ceiling effects, interitem correlations, item-total correlations, and Cronbach coefficient alpha (CCA). Rasch models (RM) examined the fit of data. Tools were reduced in items to secure robust tools fit for screening purposes. Reductions were made based on psychometrics, face validity, and content validity.

**Results:**

Tool 1 was not reduced in items; it consequently consists of 10 items. The overall fit to the RM was acceptable (Anderson conditional likelihood ratio, CLR=10.8; df=9; *P*=.29), and CCA was .67. Tool 2 was reduced from 20 to 9 items. The overall fit to a log-linear RM was acceptable (Anderson CLR=78.4, df=45, *P*=.002), and CCA was .85. Tool 3 was reduced from 23 to 5 items. The final version showed excellent fit to a log-linear RM (Anderson CLR=47.7, df=40, *P*=.19), and CCA was .90. Tool 4 was reduced from 12 to 6 items. The fit to a log-linear RM was acceptable (Anderson CLR=42.1, df=18, *P*=.001), and CCA was .59. Tool 5 was reduced from 20 to 6 items. The fit to the RM was acceptable (Anderson CLR=30.3, df=17, *P*=.02), and CCA was .94. Tool 6 was reduced from 5 to 4 items. The fit to a log-linear RM taking local dependency (LD) into account was acceptable (Anderson CLR=26.1, df=21, *P*=.20), and CCA was .91. Tool 7 was reduced from 6 to 4 items. The fit to a log-linear RM taking LD and differential item functioning into account was acceptable (Anderson CLR=23.0, df=29, *P*=.78), and CCA was .90.

**Conclusions:**

The eHLA consists of 7 short, robust scales that assess individual’s knowledge and skills related to digital literacy and health literacy.

## Introduction

Health care is transforming toward increased patient involvement with the ultimate goal of patients being able to take better care of their own health. This requires that we understand the health-related competencies that patients need to be able to handle information and actively engage in their health condition. Health literacy is one of the key concepts to achieve this. A definition from 1998 states: “Health literacy represents the cognitive and social skills which determine the motivation and ability of individuals to gain access to, understand, and use information in ways which promote and maintain good health” [1].

In accordance with this definition, early measurements of health literacy focused on the patient’s ability to read and understand health information [2,3]. This rather narrow understanding has been widened in later multidimensional definitions of health literacy that include, for example, taxonomic levels, navigation in health systems, and social interaction [4-6]. Examples of multidimensional instruments for measuring health literacy include European Health Literacy Survey (HLS-EU) and Health Literacy Questionnaire (HLQ) [4,5].

Simultaneously with the development of health literacy, increased technology use led to the definition of computer literacy or digital literacy as the understanding of necessary skills in technology use and problem-solving [7]. Digital literacy soon became relevant in the health care setting with the emergence of patient-involving digital health care services, for example, email correspondence with general practitioners and looking up health information online.

In 2006, Norman and Skinner addressed this new need to understand the users’ digital competencies in a health context. They defined electronic health (eHealth) literacy as “the ability to seek, find, understand, and appraise health information from electronic sources and apply the knowledge gained to addressing or solving a health problem” [8] and introduced the 8-item eHealth Literacy Scale (eHEALS) instrument for measuring eHealth literacy [9]. In their understanding, a user’s eHealth literacy consists of 3 contextual literacies (health literacy, computer literacy, and science literacy) and 3 analytical literacies (traditional literacy, information literacy, and media literacy) [8]. This model is referred to as the lily model, and it represents our initial understanding of the eHealth literacy concept. The model was expanded in 2009 by Chan and Kaufman, who suggested the addition of taxonomic levels for each of the 6 subliteracies, whereas another expansion by Gilstad in 2014 added contextual, cultural, and social dimensions [10-12]. Furthermore, Gilstad introduced a new definition: “eHealth literacy is the ability to identify and define a health problem, to communicate, seek, understand, appraise and apply eHealth information and welfare technologies in the cultural, social and situational frame and to use the knowledge critically in order to solve the health problem” [12].

Both Chan and Kaufman, and Gilstad elaborated on the existing concept of eHealth literacy by expanding the lily model. However, in 2015, 2 new conceptual understandings of eHealth literacy emerged.

Inspired by the HLS-EU, Bautista performed a systematic keyword clustering on definitions of health literacy, digital literacy, and eHealth literacy [5,13]. This led to a new proposal of a definition of eHealth literacy: “eHealth literacy involves the interplay of individual and social factors in the use of digital technologies to search, acquire, comprehend, appraise, communicate and apply health information in all contexts of health care with the goal of maintaining or improving the quality of life throughout the lifespan*”* [13]*.* In the same year, the authors of this study, together with a research group from Deakin University, introduced the eHealth Literacy Framework (eHLF) [14].

eHLF consists of the following 7 dimensions derived from a structured concept mapping process involving both professionals and patients: “ability to process information,” “engagement in own health,” “ability to actively engage with digital services,” “feel safe and in control,” “motivated to engage with digital services,” “access to digital services that work,” and “digital services that suit individual needs,” together presenting a multifaceted understanding of eHealth literacy [14]. Subsequently, the eHealth Literacy Questionnaire (eHLQ) was developed as an instrument for measuring eHealth literacy based on the 7 dimensions [15,16].

In this paper, we present the eHealth Literacy Assessment toolkit (eHLA) with a different approach to understand and assess eHealth literacy, compared with the conceptual development. The approach was to combine health literacy, computer and digital literacy, and information literacy in the eHLA. These 3 elements are central subliteracies in the lily model’s understanding of eHealth literacy, as well as included in the eHLF’s dimensions that describe the individual and the system interaction [8,14]. The combination allows for a deeper understanding of the competencies, knowledge, and skills that a person needs to use and adopt eHealth solutions.

This process was initiated before the development of eHLF and eHLQ, and eHLA has since continued its development in parallel with eHLQ. With eHLA, it has been a goal to develop a toolkit with the combination of test and self-assessment elements and a toolkit suitable for screening purposes in projects involving eHealth solutions.

A similar approach combining skill and self-assessment is seen in the development of the Digital Health Literacy Instrument (DHLI) introduced in January 2017 [17]. DHLI consists of 21 self-assessed items supplemented with 7 performance tasks that focus on handling digital information, mainly related to navigating the Internet and messaging health professionals [17].

Our approach to the development of eHLA was to combine tools from the more well-established fields of digital/computer literacy and health literacy in a toolkit. This meant to evaluate existing tools and identify those that could be used to improve the understanding of a person’s level of eHealth literacy in a screening situation, by being easily administered, automatically evaluated, and independent of reviewer [18].

From 2011 to 2016, the tools went through continuous development and adjustments. Tools were developed to reflect the concept of eHealth literacy, first from the understanding represented in Norman and Skinner’s work and later from the understanding represented in the eHLF. Item adjustments were made to ensure that items were robust to changes in technology and different population settings, and that tools could be used together or on their own.

The eHLA was constructed with the purpose to screen and assess the eligibility of an individual’s participation in projects involving eHealth solutions. It consists of 7 tools, 4 tools evaluate competencies related to health and 3 tools for evaluating digital aspects. The final toolkit is based on a mixture of an existing scale [19], adapted scales [2,20,21], and scales developed specifically for eHLA in a combination of self-assessment and skill-based methods.

In this study, we describe the development and the validation of the eHLA.

## Methods

### Development of the Toolkit

The eHLA was developed during the period 2011 to 2016 through continuous testing and adjustments in several iterations with the aim of having a robust and adequate toolkit. Table 1 provides an overview of the 10 tools that were included or developed and tested in the process. The initial tool candidates for eHLA were identified in 2011. A total of 5 tools were evaluated by author ON in a population of 24 diabetes patients. On the basis of these initial results, the second iteration with 4 tools was made in 2012. 

**Table 1:** Development of eHealth literacy assessment toolkit (eHLA). This table illustrates how 10 different tools were adapted or developed through 5 development phases. Seven of the 10 tools were included in the final version of the eHLA.

Tool name	Part of eHLA	Perspective	Development phases						Origin		Type
			2011	Spring 2012	Spring 2014	Spring 2015	Autumn 2015				
Powers	–	Digital	3 items (Transl^a^)	–	–	–	–	Power [19]		Self-rated	
Functional health literacy	Tool 1	Health	12 items (Transl)	12 items	12 items	12 items	10 items	Parker et al [2]		Performance	
eHEALS^b^	–	Health/digital	8 items (Transl)	–	–	–	–	Norman and Skinner [8]		Self-rated	
Technology familiarity	Tool 5	Digital	30 items (Transl)	13 items (Adj^c^ + red^d^)	20 items (Adj)	20 items (Adj)	23 items (Adj)	Hargittai et al [18]		Self-rated	
Technology confidence	Tool 6	Digital	13 items (Transl)	5 items (Adj + red)	5 items	5 items	5 items	Cho [17]		Self-rated	
COPD^e^ tool	–	Health/digital	–	1 item (Dev)	–	–	–	Original by authors		Performance	
Health literacy self-assessment/HLS^f^	Tool 2	Health	–	–	7 items (Red)	7 items	20 items (Add^g^)	Sørensen et al [5]		Self-rated	
Knowledge of health and disease	Tool 4	Health	–	–	6 items (Dev^h^)	7 items (Adj)	12 items (Adj + red)	Original by authors		Performance	
Incentives for engaging with technology	Tool 7	Digital	–	–	6 items (Dev)	6 items	6 items	Original by authors		Self-rated	
Familiarity with health and health care	Tool 3	Health	–	–	–	–	23 items (Dev)	Original by authors		Self-rated	

^a^Transl: translation from English to Danish.

^b^eHEALS: eHealth Literacy Scale.

^c^Adj: adjustments and changes to items.

^d^Red: reduction in items.

^e^COPD: chronic obstructive pulmonary disease.

^f^HLS: Health Literacy Survey.

^g^Add: items from original scale added.

^h^Dev: development of new items.

This iteration was pilot tested in a population of 7 patients with chronic obstructive pulmonary disease (COPD; Table 1) [22]. The number of items was reduced for all tools during this evaluation [22].

In 2014, 2 new scales and an existing questionnaire were added to eHLA. This third iteration was evaluated by author DF in cognitive tests (n=4) and a pilot study (n=7) [18,19].

In spring 2015, the fourth iteration with minor adjustments was tested in 8 cognitive interviews and validated in a convenience sample with 187 respondents who completed a digital version of the toolkit [23]. In autumn 2015, 5 cognitive interviews led to minor adjustments of the health-related tools and the addition of a tool exploring familiarity with health and health care.

### Cognitive Interviews

Iterations of eHLA were continuously tested using cognitive interviews to make sure that the items in each tool were understood as intended [22,24]. In each cognitive interview, the respondent was provided with a paper version of eHLA. The respondent was then asked to fill out the questionnaire while the interviewer carefully observed and noted items that caused problems or hesitation in the respondent. After the questionnaire had been completed, the interviewer and the respondent would go through the questionnaire and focus on items difficult to the respondent. The interviewer would ask “What were you thinking about when you were answering that question?” This process elicited the cognitive process behind the answers. A prompt was used if needed: “Why did you select that response option?” The aim of this process was to eliminate items with ambiguity, items with strong relation to prior experience, and items favoring larger population groups such as females or people with heart conditions.

### The 7 Tools of eHealth Literacy Assessment Toolkit

A total of 7 tools, 4 health-related and 3 digitally-related, comprise the validation version of eHLA (compare to Table 1). The 4 health-related tools are functional health literacy (tool 1), self-assessed health literacy (tool 2), familiarity with health and health care (tool 3), and knowledge of health and disease (tool 4). The 3 digitally-related tools are familiarity with technology (tool 5), technology confidence (tool 6), and incentives for engaging with technology (tool 7). eHLA is presented in Danish in Multimedia Appendix 1 and with an English excerpt of items in Multimedia Appendix 2.

#### Health-Related Tools

##### Tool 1—Functional Health Literacy

Tool 1 is inspired by Test of Functional Health Literacy in Adults [2]. This tool is a performance test that tests a respondent’s ability to read, write, understand, and perform a simple calculation in a health context. The tool is structured as a performance test in which respondents are given a text excerpt from a paracetamol leaflet. In each of the 10 items, a word (8 items) or a number (2 items) has been replaced with a blank. The respondent fills out the blank by underlining 1 of the 4 different response options. One option is correct and 3 are incorrect.

Tool 1 was included in eHLA from first iteration in 2011. Cognitive interviews and pilot tests showed that the items were too easy with almost no incorrect answers. In 2012, as a result, response options for all items were adjusted to increase difficulty levels. In the initial validation in 2015, 2 items yielded no incorrect answers from respondents and contributed to a severe tool ceiling effect. Consequently, these 2 items were removed.

The validation version of tool 1 consisted of 10 items. Results for each participant were calculated as the sum of correct answers, with a minimum score of 0 and a maximum of 10 points.

##### Tool 2—Health Literacy Self-Assessment

Tool 2 is a shortened version of the HLS-EU-Q47 questionnaire measuring self-reported health literacy [19]. All items were used directly and with no change of wording. Tool 2 aims to give a rough measure of health literacy as defined by the HLS-EU framework matrix, which consists of 3 areas—health care, disease prevention, and health promotion—under which 4 taxonomic levels are defined: find, understand, appraise, and apply. All items can be mapped into one of the cells in the matrix and are formulated as a question of how easy a task is to perform with responses given on a scale from 1 to 4, ranging from very difficult to very easy.

This tool was initially added to eHLA in 2014. To cover the increasing difficulty of the taxonomic levels, items handling the same situation were chosen, covering an emergency situation and a situation of handling information from the media. In total, 7 items out of the 47 from HLS-EU-Q47 were included in the tool.

In 2015, an additional 13 items were added to ensure that the validation version comprised both the items selected by us as well as all items from the 16-item short version of HLS-EU-Q47, the HLS-Q16 [19].

The validation version of eHLA tool 2 consisted of 20 items with a scoring range of 20 to 80 points in the validation version.

##### Tool 3—Familiarity With Health and Health Care

Tool 3 was created specifically for eHLA by 3 of the authors (DF, LK, and AK). The scale is inspired by the work of Hargittai et al who used the familiarity scale in a digital context [21]. The aim of the self-reported tool is to assess familiarity with the health care system and typical terminology used in the health care. Respondents are asked to rate a number of health care–related terms and concepts on a scale from 1 to 4, ranging from “not at all familiar” to “completely familiar.”

Familiarity with health and health care was added to eHLA in autumn 2015 and consequently did not go through prevalidation. In cognitive interviews, the respondents provided positive feedback on the format, which they found easy and accessible.

The validation version consisted of 23 items; thus, scores range from 23 to 92.

##### Tool 4—Knowledge of Health and Health Care

Tool 4 was developed specifically for eHLA in spring 2014. We wished to include a performance test that tested knowledge of health and health care. The tool was designed as a multiple-choice quiz with questions. For each question, there are 4 response options: 1 correct answer (2 points), 2 incorrect answers (0 points), and one “I would consult with someone else” option (1 point). Thus, partial credit is given to respondents choosing the latter option.

After 2 rounds of cognitive interviews in 2015, thorough changes were made to the items to avoid favoring specific patient groups and to better distinguish lower levels of knowledge from higher levels.

The validation version of tool 4 consisted of 12 items with a possible sum score ranging from 0 to 24 points.

#### Introduction to the Digitally-Related Tools in eHealth Literacy Assessment Toolkit

The digitally-related tools are meant to cover a range of technologies, but when the word “technology” was used in cognitive interviews, respondents found it difficult to relate to the items. Several tests showed that the best solution was to use the word “computer” in all 3 tools and then add a short introduction that explains that the word computer is used as a term that covers all technologies used in everyday life.

##### Tool 5—Familiarity With Technology

Tool 5 assesses familiarity with technology based on the work of Hargittai et al, who showed this method to be a valid proxy for digital skills [21]. The concept of the original scale remains, but the items have been changed and adapted to the context of eHLA. New items were selected from the following criteria: (1) they should work in both Danish and English, (2) they should not only relate to a single type of technology, and (3) they should be robust over time. The aim of tool 5 is to estimate familiarity and the respondent’s knowledge level.

In 2012, the tool was reduced to 13 items to better reflect technology as a wider term than computers only. In 2014 and 2015, it was adjusted to include a broader difficulty spectrum.

The validation version of tool 5 contained 20 items with varying difficulty. Each item was rated from 1 to 4—1 being “not at all familiar” and 4 being “completely familiar,” with a potential range of sum scores from 20 to 80.

##### Tool 6—Technology Confidence

Tool 6 was inspired by Cho et al [20]. This tool is used to investigate how confident a respondent feels when using technology in general. The tool is based on a self-reported approach in which respondents are asked to rate how confident they feel performing the task stated in the item.

In 2012, the tool was adjusted to better include technology as a wider term than computers only.

The validation version of tool 6 in eHLA consisted of 5 items. The response options were on a scale from 1 to 4, with 1 being very unconfident and 4 being very confident. Sum score ranged from 5 to 20.

##### Tool 7—Incentives for Engaging With Technology

In 2014, tool 7 was developed specifically for eHLA as a tool to investigate motivation for engaging with technology. The tool was constructed after the introduction of the eHLF to include perspectives on motivation to engage with digital services. The tool is a self-reported questionnaire with items based on statements from the concept mapping process that formed dimension 5 of the eHLF [14].

The validation version of this tool contained 6 items with response options from 1 to 4, with 1 being “completely disagree” and 4 being “completely agree.”

### Other Tools

A total of 3 other tools were tested but later removed from the eHLA before the validation.

eHEALS [9] was included in the initial development phase as this, at the time, was the only available tool for measuring eHealth literacy. However, it was removed because of limited applicability to the Danish context, primarily because the term “health resources on the Internet” is used in several items. In particular, the word “resources” cannot be translated to a simple concept related to a person’s use of the health care system in Denmark.

A tool consisting of 3 questions for identifying patients with limited functional health literacy suggested by Powers et al [25] showed limited ability to discriminate the participants’ literacy levels and was subsequently omitted.

A COPD tool was developed and added to the second iteration of eHLA to assess patients’ critical health and literacy levels, but it was later removed to avoid disease-specific tools. Furthermore, replies were given as free text, which complicated the analyses [22].

### Intertool Correlations

A total of 4 tools are used to assess health-related literacy, and 3 tools assess digitally-related literacy. Intertool correlations were calculated to improve the understanding of how the tools supplement and possibly overlap each other. The expectation was that the 7 tools measured different aspects of eHealth literacy, but with higher correlations within each of the fields for health and digital literacy.

### Final Construction of eHealth Literacy Assessment Toolkit

#### Data Collection for Validation of the eHealth Literacy Assessment Toolkit

Data collection for the validation was conducted from October to December 2015. The eHLA was distributed together with a validation version of the eHLQ [16], and a sociodemographic questionnaire included age, gender, education, self-rated health, other chronic diseases, and nationality. The complete questionnaire consisted of 96 items in eHLA, 58 items in eHLQ, and 6 sociodemographic questions.

Respondents were recruited from an outpatient clinic at Gentofte Hospital, north of Copenhagen, Denmark, and from a general population sample (recruited during visits at, eg, libraries, work places, and sports events). All questionnaires in the outpatient clinic were distributed in paper versions, and respondents who did not have time to finish the questionnaire onsite were given a prepaid envelope they could return the questionnaire in. One of the authors (AK) and 4 student assistants from Institute of Sociology at University of Copenhagen recruited respondents by visiting workplaces, sport events, libraries, and nursing homes. Data collection was carried out as in the outpatient clinic, but with an additional option of filling it out digitally. The recruitment method does not allow for an analysis of those who chose not to respond.

### Statistics

Initial data analysis evaluated floor and ceiling effects, interitem correlations, item-total correlations, and calculation of Cronbach coefficient alpha (CCA) [26]. After a potential reduction in the number of items, further analyses examined fit of the data to the Rasch model (RM) [27,28] and evaluated differential item functioning (DIF) with regard to age and gender [29]. We evaluated the overall fit of a subscale to the RM using the Anderson conditional likelihood ratio (CLR) test [30], tested the fit of individual items using comparison of observed and expected item-rest score correlation [31], and evaluated DIF and local dependency (LD; [32]) using the Q3 index [33] log-linear RM tests [34]. In additional analyses, we evaluated the overall fit and the item fit in a log-linear RM where LD was added.

Pearson correlation coefficients were used to estimate correlations between the 7 tools.

Reductions and adjustments to items in this final validation round were done in group session with the authors, and all decisions were made based on statistical results, item content, and information from cognitive interviews.

### Ethics

According to Danish law, when survey-based studies are undertaken in accordance with the Helsinki Declaration, specific approval by an ethics committee and written informed consent is not required. Potential respondents were provided with information about the survey and its purpose, including that participation was voluntary. The completion of the survey by participants was then considered to be implied consent.

## Results

### Data Collection for Validation of the eHealth Literacy Assessment Toolkit

A total of 100 questionnaires were collected from outpatients (paper and pen) and 375 were collected from the community (328 paper and pen and 47 digitally). The final validation sample consisted of 475 questionnaires. See sociodemographics in Table 2.

The final version of eHLA is presented in its original Danish version in Multimedia Appendix 1, and a translated English excerpt is presented in Multimedia Appendix 2.

Results of the validation are summarized in Table 3.

**Table 2:** Sociodemographics for the respondents (N=475).

Sociodemographics			n (%)
**Age in years**			
	18-35	147 (30.9)	
	36-60	174 (36.6)	
	60+	133 (28.0)	
**Gender**			
	Male	213 (44.8)	
	Female	245 (51.6)	
**Education**			
	No education	38 (8.0)	
	Short education	179 (37.7)	
	Long education	224 (47.2)	
**Self-rated health**			
	Excellent	196 (41.3)	
	Good	186 (39.2)	
	Bad	78 (16.4)	
**Chronic conditions**			
	Yes	189 (39.8)	
	No	269 (56.6)	

**Table 3:** Summary of the validation of the 7 eHealth literacy assessment tools.

Tool and item			Item-rest score correlation								
		Observed		Rasch model				Log-linear Rasch model			
				Expected		*P* value		Expected		*P* value	
**Functional health literacy (tool 1)**													
	1-1	.65		.76		.39		–		–	
	1-2	.80		.81		.95		–		–	
	1-3	.69		.68		.89		–		–	
	1-4	.46		.75		.02		–		–	
	1-5	.59		.65		.35		–		–	
	1-6	.65		.65		.98		–		–	
	1-7	.72		.65		.30		–		–	
	1-8	.85		.70		.10		–		–	
	1-9	.66		.77		.38		–		–	
	1-10	.74		.72		.80		–		–	
**Self-assessed health literacy (tool 2)**													
	2-1	.64		.65		.88		.66		.66	
	2-2	.64		.62		.61		.65		.79	
	2-3	.66		.64		.63		.69		.51	
	2-4	.77		.64		.01		.75		.53	
	2-5	.63		.63		.99		.64		.86	
	2-6	.58		.63		.18		.59		.77	
	2-7	.63		.64		.66		.60		.54	
	2-8	.64		.64		.99		.60		.32	
	2-9	.69		.63		.12		.59		.01	
**Familiarity with health and health care (tool 3)**													
	3-1	.76		.78		.28		.73		.43	
	3-2	.73		.78		.02		.73		.84	
	3-3	.82		.78		.10		.80		.57	
	3-4	.85		.78		.001		.86		.87	
	3-5	.80		.78		.46		.80		.82	
**Knowledge of health care (tool 4)**													
	4-1	.35		.44		.06		.38		.61	
	4-2	.66		.36		<.001		.59		.21	
	4-3	.54		.47		.17		.52		.72	
	4-4	.42		.56		.008		.51		.11	
	4-5	.52		.38		.02		.44		.18	
	4-6	.37		.40		.65		.33		.42	
**Familiarity with technology (tool 5)**													
	5-1	.85		.86		.80		–		–	
	5-2	.85		.87		.39		–		–	
	5-3	.89		.86		.23		–		–	
	5-4	.90		.87		.09		–		–	
	5-5	.85		.87		.22		–		–	
	5-6	.88		.86		.37		–		–	
**Technology confidence (tool 6)**													
	6-1	.91		.87		.08		.92		.56	
	6-2	.90		.88		.22		.90		.85	
	6-3	.87		.87		.90		.82		.05	
	6-4	.82		.87		.002		.85		.13	
**Incentives for engaging with technology (tool 7)**														
	7-1	.83		.86		.21		.83		.93	
	7-2	.86		.85		.65		.85		.67	
	7-3	.87		.85		.33		.86		.81	
	7-4	.88		.85		.10		.87		.76	

### Tool 1—Functional Health Literacy

Tool 1 was completed by 404 participants in the validation. The distribution of the score was very skewed. A total of 273 respondents (67.6%) had a maximum score of 10 points, 93 (23.0%) respondents got 9 points, and 28 (9.5%) respondents got 8 points or less.

Interitem correlations ranged from .03 to .39, with item-total correlations being generally low ranging from .40 to .55. No evidence of LD or DIF was disclosed. The overall fit to the RM was acceptable (Anderson CLR=10.8, df=9, *P*=.29) and CCA was .67.

No reduction in items was performed, and consequently, the final version of eHLA tool 1 consists of 10 items.

### Tool 2—Health Literacy Self-Assessment

In the first reduction, 4 of the 20 items were removed, 2 because of evidence of DIF. In the second round of reduction, Rasch analyses and considerations about the HLS matrix with the aim of coverage of the framework informed further removal of 7 items. This yielded a 9-item subscale with construct and content validity. The overall fit to a log-linear RM was acceptable (Anderson CLR=78.4, df=45, *P*=.002) and CCA was .85.

### Tool 3—Familiarity With Health and Health Care

In the initial analysis, item-total correlations ranged from .54 to .79. In the first round of reductions, 5 items were removed based on a combination of content and data analysis.

In the second round of reductions, a total of 13 items were removed based on evaluation of Rasch item fit statistics, evidence of DIF, and content validity considerations. As an example, the item “emergency room” was found to have the lowest item-total correlation, and several authors expressed concerns that the item and concept of an emergency room will change and that there is a risk that the item may not be meaningful in few years because of organizational changes. Furthermore, the items “myocardial infarction” and “gastroenteritis” were removed as they fitted the RM poorly. Evidence of LD for the item pairs (1,2), (3,4), and (4,5) was disclosed.

The final version of tool 3 consisted of 5 items and showed excellent fit to a log-linear RM (Anderson CLR=47.7, df=40, *P*=.19) and CCA was .90.

### Tool 4—Knowledge of Health and Disease

Five items were removed in the first round of reduction. Reductions were based on a combination of content, item-total correlations, tests of DIF, and Rasch analysis. Interitem correlations ranged from .08 to .45, and item-total correlations ranged from .48 to .70.

In the second round of reductions, one more item was removed. The item “meningitis” showed significant DIF with regard to age and gender and had the lowest item-total correlation. This might indicate that knowledge about meningitis is more extensive among people with children and especially mothers. The Rasch analysis disclosed evidence of LD for the item pairs (2,3) and (2,5).

The final, validated version of tool 4 consists of 6 items. The fit to a log-linear RM was acceptable (Anderson CLR=42.1, df=18, *P*=.001) and CCA was .59.

### Tool 5—Familiarity With Technology

The cognitive interviews underlined the importance of displaying the words in each item in both Danish and English, as respondents would sometimes recognize only one of the languages.

In the validation data for tool 5, item-total correlations ranged from .59 to .89. Reductions were performed based on DIF, content, and Rasch analysis. For example, the item “macro” was removed as it contained an ambiguity content-wise, which authors had not been aware of earlier.

**Table 4:** Pearson correlation coefficients between tools.

eHLA^a^ tools		Pearson correlation coefficient						
		Health-related tools				Digitally-related tools		
		Tool 1	Tool 2	Tool 3	Tool 4	Tool 5	Tool 6	Tool 7
**Health-related tools**								
	Tool 1	1.00	–	–	–	–	–	–
	Tool 2	.18	1.00	–	–	–	–	–
	Tool 3	.21	.41	1.00	–	–	–	–
	Tool 4	.40	.30	.30	1.00	–	–	–
**Digital tools**								
	Tool 5	.32	.36	.40	.20	1.00	–	–
	Tool 6	.30	.38	.33	.17	.83	1.00	–
	Tool 7	.22	.30	.28	.14	.71	.76	1.00

^a^eHLA: eHealth Literacy Assessment toolkit.

This was also reflected in Rasch analysis. The item “turn on the computer” was removed because of a severe ceiling effect, where 387 (87%) respondents replied “completely familiar.”

The final version of tool 5 contains 6 items. The fit to the RM was acceptable (Anderson CLR=30.3, df=17, *P*=.02) and CCA was .94.

### Tool 6—Technology Confidence

Validation data showed that 211 (47%) respondents had a sum score of 19 or 20 points, reporting a maximum score. Interitem correlations ranged from .63 to .79. Item-total correlations ranged from .87 to .91. After Rasch analysis and tests for DIF, the item “to open and save a file” was removed.

The Rasch analysis indicated LD between items 6a and 6b in this tool.

The final tool 6 consists of 4 items. The fit to a log-linear RM taking LD into account was acceptable (Anderson CLR=26.1, df=21, *P*=.20) and CCA was .91.

### Tool 7—Incentives for Engaging With Technology

Validation data showed right-skewed data. Interitem correlations were in the range of .39 to .83, and item-total correlations were in the range of .67 to .86. DIF on age and gender showed significant, but not very strong, correlations to 3 of the items.

A total of 2 items were excluded from the tool based on Rasch analyses and content evaluation. The item “computers bring us closer together” performed poorly in item-total correlations, and the content was assessed to be opinion-based and not necessarily an indicator of motivation. The items are intended to explore motivation related to the respondent’s own experience. Consequently, the item “computers can be useful in everyday life” was excluded mainly for its ambiguity and lack of relatedness to the respondent’s own situation. The Rasch analysis disclosed evidence of LD for the item pairs (1,2) and (3,4) and DIF for item 2 with regard to gender.

The final version of tool 7 consists of 4 items. The fit to a log-linear RM taking LD and DIF into account was acceptable (Anderson CLR=23.0, df=29, *P*=.78) and CCA was .90.

The validation is summarized in Table 3.

### Intertool Correlations

Correlations between all 7 tools were estimated using Pearson correlation coefficients (see Table 4). The health-related tools have correlations ranging from .18 (tools 1 and 2) to .41 (tools 2 and 3). Results from the Pearson correlation coefficients show that the 3 digitally-related tools (tools 5, 6, and 7) are strongly correlated with scores ranging from .70 (tools 5 and 7) to .83 (tools 5 and 6).

Familiarity with health and health care (tool 3) and technology familiarity (tool 5) have the highest intertool correlation (.40) across digitally- and health-related tools.

## Discussion

### The eHealth Literacy Assessment Toolkit

The eHLA provides the means for gaining insight into people’s health-related literacy as well as their confidence, familiarity, and motivation related to digital solutions. This toolkit consists of 7 tools that validly measure constructs with a satisfactory fit to log-linear RMs, thus displaying essential validity and objectivity [35].

### Reflection on Method

The development of the eHLA was initiated to be able to swiftly and robustly evaluate a person’s eHealth literacy level. The screening format influenced the design and development process in terms of (1) restrictions of total item number and (2) tools should be compatible as self-administration.

Initially, existing tools were used. However, the tested tools conflicted with the screening format, and it instigated the development of new tools. This development process has led to deeper understanding of the aspects of eHealth literacy. However, the development of new tools is an extensive task, which is not to be recommended where valid and useful tools preexist.

### The 7 Tools of eHealth Literacy Assessment Toolkit

Results of the performance-based functional health literacy tool (tool 1) were affected by a low number of respondents with low scores. This may be due to our population sample in the validation, as a study has shown that a Danish validated version of the Test of Functional Health literacy in Adults identified 26% with inadequate levels of functional health literacy in a population of COPD patients (mean age 68.7 years) [36]. This may suggest that eHLA’s tool 1 is more relevant to be included in studies among older people with chronic health conditions.

Furthermore, tool 1 (functional health literacy) shows higher correlations to the digitally-related tools than to 2 of the health-related tools (tools 2 and 3). Tool 1 tests the ability to read, write, understand, and calculate in a health context, and these skills may be more similar to information literacy and basic literacy as it is used in relation to technology.

With eHLA’s tool 2, comes the introduction of a 9-item short version of the HLS-EU-Q47 questionnaire [19]. The self-rated health literacy tool performed well in statistical tests. The initial validation version consisted of a selection of 20 items from the HLS-EU. In general, of these 20 items, the items in the higher taxonomic levels (appraise and apply) performed worse in statistical tests than the ones in lower levels (find and understand). A study by Neter and Brainin suggests that appraise and apply should be merged into one category [37]. These findings are supported by the results of our analysis, where the final 9 items of tool 2 cover all taxonomic levels in the HLS-EU matrix, when the appraise and apply categories are merged. Consequently, we estimate the 9-item version to be a reliable and validated short version of the HLS-EU-Q47.

Tool 3 and tool 4 were developed for eHLA, and the items in both tools address specific health elements, with tool 3 being self-assessed and tool 4 being a performance test. Tools 1 and 2 assess health competencies related to information processing and navigation in the health services, whereas tools 3 and 4 focus on an individual’s familiarity and knowledge of health and health care. The validated versions of tools 3 and 4 are robust measures of health and health care familiarity and knowledge, without items favoring specific patient populations.

It is worth noting that between the health-related tools, the 2 performance tests have higher correlations to each other (tools 1 and 4), and the 2 self-assessment tools have higher correlations to each other. This might indicate a difference between the self-assessment and the performance approach.

The Pearson correlation coefficient test across the 7 tools showed very high correlations between the digitally-related tools (tools 5, 6, and 7) compared with the correlations between the health-related tools. This might indicate that the 3 tools cover some of the same constructs. Consequently, an eHealth project might only need to administer 1 of the 3 digitally-related tools, which can be chosen depending on whether there is a greater need for investigating motivation, the feeling of security, or technology familiarity.

### Limitations

Like eHEALS, eHLA does not include the external factors suggested by Gilstad [12], such as social and contextual literacies.

It is a limitation of the toolkit that it does not include a tool to assess the communicative and social aspects of digital literacy in a Web 2.0 context. Existing tools in the area are scarce, and it is still very diffuse what to measure.

Recruitment was conducted in a general population sample as well as in an outpatient clinic. The recruitment was efficient; however, it is a limitation of the study that dropout analyses could not be carried out because of data collection design.

### eHealth Literacy Assessment Toolkit Versus eHealth Literacy Scale, Digital Health Literacy Instrument, and eHealth Literacy Questionnaire

Norman and Skinner’s eHEALS is based on the lily model with its 6 subliteracies [8,9]. However, eHEALS results are calculated as a sum score, and although there might be 2 or more constructs in the items [37,38], the sum score does not reflect eHealth literacy as a multidimensional construct. eHLA contains elements from all 6 subliteracies in the lily model, and it distinguishes between health and digital literacy. Validation showed that eHLA’s tools measure different aspects of digital health. If digital literacy and health literacy are considered the core elements of the lily model, it is possible that tools from eHLA elaborate on key element from the lily model [39,40].

A study by van der Vaart et al showed that eHEALS scores were poorly correlated with actual Internet use [39]. Similar to eHEALS, the eHLA is mainly based on self-assessment with only tools 1 and 4 being health-related tests (yet self-reported tests), and it is to be considered a limitation that no performance tools were identified as a fit for the screening format used in the eHLA. However, with eHLA being a toolkit for screening participants in eHealth projects, we argue that the self-assessed skill will be of importance to the actual use of the technology. A study showed computer use to have a positive effect on computer self-confidence and computer-related attitudes. Furthermore, these 2 factors had a positive effect on computer knowledge [41].

Future studies will have to investigate the correlation between eHLA scores and actual use of eHealth technology by participants in eHealth projects.

DHLI primarily measures digital skills in a health context [17], whereas eHLA assesses digitally- and health-related measures separately. Although there are overlaps between, for example, eHLA’s technology familiarity scale and DHLI’s operational skills construct, it is possible that tools from eHLA and subscales from DHLI can be combined to suit the need of specific eHealth projects. Further studies would have to be conducted to explore possible combinations.

eHLA and the eHLQ were developed simultaneously. The eHLQ items are based on the eHLF’s 7 dimensions, and its 7 scales provide detailed insight on an individual’s or a population’s eHealth literacy levels. The eHLA tools have more easy-to-understand and content-specific items that make the toolkit useful in practice settings. The tools in eHLA assess health literacy and digital literacy, and although eHLQ also contain elements of these, eHLA’s tools are comprehensive with a combination of self-assessment and test of skills. These advantages make eHLA suitable for use in screening purposes.

eHLA’s 7 tools all performed adequately in the statistical validation tests. The construction and validation of eHLA allow future eHealth projects to use the toolkit both as a whole or with a selection of the 7 tools.

Future research should look into validation of eHLA as a tool for screening and preferably test the toolkit in a set of eHealth projects to determine how the tools work as predictors of actual skills and benefit—together, on their own, or in combination with, for example, DHLI. Furthermore, we currently investigate how eHLA correlates to classic sociodemographic factors, as well as how eHealth literacy can supplement assessments of health literacy and empowerment.

### Conclusions

eHLA is a validated, robust toolkit consisting of 7 tools that each highlight a specific component of the competencies and resources in eHealth literacy that are necessary for a person to achieve the optimal outcome in projects involving eHealth solutions.

eHLA is suitable for studies that need tools for screening participants’ knowledge and skills related to eHealth literacy.

## Appendix

Multimedia Appendix 1 The eHealth Literacy Assessment toolkit in Danish.

Multimedia Appendix 2 The English excerpt of the eHealth Literacy Assessment toolkit.

## Abbreviations

CCA: Cronbach coefficient alpha
CLR: conditional likelihood ratio
COPD: chronic obstructive pulmonary disease
DHLI: Digital Health Literacy Instrument
DIF: differential item functioning
eHealth: electronic health
eHEALS: eHealth Literacy Scale
eHLA: eHealth Literacy Assessment toolkit
eHLF: eHealth Literacy Framework
eHLQ: eHealth Literacy Questionnaire
HLS-EU: European Health Literacy Survey
LD: local dependency
RM: Rasch model
