# Supplementary material for: Assessing Competencies Needed to Engage With Digital Health Services: Development of the eHealth Literacy Assessment Toolkit
Source: J Med Internet Res. 2018 May 10;20(5):e178. doi: 10.2196/jmir.8347 (PMC5968212; doi:10.2196/jmir.8347)
Supplement: Multimedia Appendix 1 [file jmir_v20i5e178_app1.pdf]

eHealth Literacy Assessment Toolkit - eHLA

1. Fortrolighed med medicinvejledning

Forestil dig dette er en indlægsseddel vedrørende Panodil-tabletter 500 mg.
Panodil er det samme som Pamol, Pinex og Paracetamol.
Udfyld de tomme felter med A, B, C eller D, afhængigt af hvilket ord du mener mangler i sætningen.

Du kan _______ Panodil uden recept.

A. se
B. få
C. er
D. gå

Panodil virker ____________________ og febernedsættende.

A. smertestillende
B. smertestimulerende
C. smertegrænse
D. smørende

Hvis din læge har ______________ Panodil til dig, skal du altid følge lægens anvisning.

A. ordentlig
B. ordineret
C. orkestret
D. ordstyrer

Er du i tvivl, så spørg lægen eller på _______________.

A. biblioteket
B. applet
C. apoteket
D. modkrav

Den sædvanlige dosis for voksne er 2 tabletter a' 500 mg (i alt________ mg) 3-4 gange dagligt

A. 250
B. 1000
C. 3000
D. 4000

og højst ______ tabletter (i alt 4000 mg) dagligt.

A. 2
B. 4
C. 6
D. 8

Valideringsudgave, Københavns Universitet. Copyright 2015.

I enkelte tilfælde kan 1 tablet på 500 mg 3-4 gange dagligt være tilstrækkeligt.

Dosis til børn afhænger af barnets vægt. Børn må få 50 mg/kg/døgn fordelt på 3-4 doser. Hvis barnet f.eks. vejer 30 kg vil dosis maksimalt være _________ mg i døgnet.

A. 500
B. 1000
C. 1500
D. 3000

Kontakt lægen, skadestuen eller apoteket, hvis du har taget flere Panodil-tabletter, end der står i denne information, eller flere end lægen har foreskrevet. Tag pakningen med.

En ___________ dosis Panodil end den anbefalede er farlig og kan give langvarige skader.

A. sødere
B. mindre
C. større
D. mængde

Det kan ødelægge leveren og i nogle tilfælde også nyrerne, bugspytkirtlen og knoglemarven.

Det er vigtigt, at du søger ____________ så hurtigt som muligt ved mistanke om overdosering.

A. væk
B. læge
C. tandlæge
D. læbe

Symptomer på overdosering kan være kvalme, opkastninger, ømhed/smerter i maven, utilpashed, gulsot og misfarvning af urin og afføring.

I værste fald vil du efter ca. 3 døgn miste ___________________ og dø af leversvigt.

A. bevisførelsen
B. benovelsen
C. beslutsomheden
D. bevidstheden

2. Adgang til informationer om sundhed og sundhedstjenester

På en skala fra meget svært til meget let, hvor let vil du så sige, det er at:

På en skala fra meget svært til meget let, hvor let vil du så sige, det er at:	Meget svært	Svært	Let	Meget let	
…finde information om behandling af sygdomme, du vil vide mere om?	❑	❑	❑	❑	Healthcare/ Access information
…finde ud af, hvor du kan få professionel hjælp, når du er syg? (fx af en læge, på apoteket, af en psykolog)	❑	❑	❑	❑	Healthcare/ Access information
…forstå, hvad din læge siger til dig?	❑	❑	❑	❑	Healthcare/ Understand information
…følge vejledning fra din læge eller fra apoteket?	❑	❑	❑	❑	Healthcare/ Apply information
…finde information om, hvad man kan gøre ved psykiske problemer som stress og depression?	❑	❑	❑	❑	Disease prevention/ Access information
…beslutte på basis af information i medierne, hvordan du vil beskytte dig selv mod sygdom? (fx aviser, brochurer, internettet eller andre medier)	❑	❑	❑	❑	Disease prevention/ Apply information
…finde oplysninger om vaner, der er gode for din psykiske trivsel? (fx meditation, motion, gåture osv.)	❑	❑	❑	❑	Health promotion/ Access information
…forstå råd om sundhed fra familie eller venner?	❑	❑	❑	❑	Health promotion/ Understand information
…vurdere, hvordan dine daglige vaner har indflydelse på din sundhed? (fx drikke- og spisevaner, motion osv.)	❑	❑	❑	❑	Health promotion/ Appraise information

Valideringsudgave, Københavns Universitet. Copyright 2015.

3. Hvor stort et kendskab har du til følgende inden for sundhed og sygdom?

Vurdér på en skala fra intet kendskab til fuldt kendskab, hvor stort et kendskab du har til følgende.

**Hvor stort et kendskab har du til:**	**Intet kendskab**			**Fuldt kendskab**
	**1**	**2**	**3**	**4**
Rehabilitering	❑	❑	❑	❑
Milt	❑	❑	❑	❑
Abstinenser	❑	❑	❑	❑
Respirator	❑	❑	❑	❑
Autisme	❑	❑	❑	❑

Valideringsudgave, Københavns Universitet. Copyright 2015.

4. Spørgsmål om sundhed og sygdom

Vælg den svarmulighed, som du mener er den rigtige til hvert af de tolv spørgsmål om krop, sundhed og sygdom.

1) Når du modtager et blodprøvesvar, hvad er hæmoglobin så et udtryk for?
   a) ❑ Din blodtype
   b) ❑ Dine blodplader
   c) ❑ Din blodprocent
   d) ❑ Jeg vil spørge en anden, da jeg er usikker

2) Hvad betyder fraktur?
   a) ❑ Opkastning
   b) ❑ Knoglebrud
   c) ❑ Nyresvigt
   d) ❑ Jeg vil spørge en anden, da jeg er usikker

3) Hvilket andet ord kan man bruge i stedet for ordet ”parese”?
   a) ❑ Lammelse
   b) ❑ Befrugtning
   c) ❑ Skalpel
   d) ❑ Jeg vil spørge en anden, da jeg er usikker

4) Nefrologi er læren om
   a) ❑ Leversygdomme
   b) ❑ Nervesygdomme
   c) ❑ Nyresygdomme
   d) ❑ Jeg vil spørge en anden, da jeg er usikker

5) Hvad er en af leverens hovedfunktioner?
   a) ❑ Afgiftning af blodet
   b) ❑ Iltning af blodet
   c) ❑ Produktion af urin
   d) ❑ Jeg vil spørge en anden, da jeg er usikker

6) Hvad er en af bugspytkirtlens hovedfunktioner?
   a) ❑ At ilte blodet
   b) ❑ At producere mavesyre
   c) ❑ At producere insulin
   d) ❑ Jeg vil spørge en anden, da jeg er usikker

De sidste tre skemaer handler brug af elektroniske enheder som computer, tablet (f.eks. iPad), smartphone, smart TV eller lignende.

Hvis du bruger andre elektronisk enheder end en computer, så tag udgangspunkt i den elektroniske enhed, som du bruger mest i din dagligdag, når du svarer på skemaerne.

5. Hvor fortrolig er du med computere?

Vurdér på en skala fra slet ikke fortrolig til helt fortrolig.
(Som støtte til dig, er den engelske oversættelse med i parentes.)

**Hvor fortrolig er du med:**	**Slet ikke fortrolig**		**Helt fortrolig**	
	**1**	**2**	**3**	**4**
Tastatur *(keyboard)*	❑	❑	❑	❑
Indstillinger (*settings)*	❑	❑	❑	❑
Kopier og indsæt (*Copy paste)*	❑	❑	❑	❑
Trådløst netværk (*Wifi)*	❑	❑	❑	❑
Styresystem, fx Windows *(operating system)*	❑	❑	❑	❑
Brugernavn (*username)*	❑	❑	❑	❑

Valideringsudgave, Københavns Universitet. Copyright 2015.

6. Din brug af computer

Vurdér på en skala fra helt usikker til helt sikker din brug af computer.

Hvor sikker føler du dig i …?	Helt usikker		Helt sikker	
	1	2	3	4
At bruge en computer i det hele taget?	❑	❑	❑	❑
At bruge en anden computer end din egen?	❑	❑	❑	❑
At finde informationer på internettet?	❑	❑	❑	❑
At benytte touchscreen?	❑	❑	❑	❑

7. Din oplevelse af computere

Vurdér på en skala fra helt uenig til helt enig.

Hvor enig eller uenig er du i følgende udsagn:	Helt uenig			Helt enig
	1	2	3	4
Jeg interesserer mig for at bruge computer.	❑	❑	❑	❑
Jeg er glad for min computer.	❑	❑	❑	❑
Jeg er ikke bange for at afprøve nye funktioner på computere.	❑	❑	❑	❑
Jeg er åben for nye muligheder for brug af computere.	❑	❑	❑	❑
