# Supplementary material for: Assessing Competencies Needed to Engage With Digital Health Services: Development of the eHealth Literacy Assessment Toolkit
Source: J Med Internet Res. 2018 May 10;20(5):e178. doi: 10.2196/jmir.8347 (PMC5968212; doi:10.2196/jmir.8347)
Supplement: Multimedia Appendix 2 [file jmir_v20i5e178_app2.pdf]

eHealth Literacy Assessment Toolkit - eHLA

Tool 1. Functional health literacy

Imagine this is a patient information leaflet on 500mg paracetamol tablets (Panodil). Fill in the blanks by choosing from the four options below each blank.

Panodil is a painkiller and also ________ your temperature when you have a fever.

A. reuses
B. increases
C. rescues
D. reduces

The maximum dose for children is 50 mg/kg/day divided in 3-4 doses per day. E.g. if the child weighs 30 kg, the maximum dose is _________ mg a day.

A. 500
B. 1000
C. 1500
D. 3000

Tool 2. Health literacy self-assessment

(eHLA item number / original item number in HLS-Q47)

On a scale from very easy to very difficult, how easy would you say it is to:		Very difficult	Difficult	Easy	Very easy
**3/2**	Find information on treatments of illnesses that concern you?	❑	❑	❑	❑
**5/16**	Follow instructions from your doctor or pharmacist?	❑	❑	❑	❑
**6/37**	Understand advice on health from family members or friends?	❑	❑	❑	❑

Tool 3. Familiarity with health and healthcare

Rate on a scale from not at all familiar to completely familiar.

**How familiar are you with the following items:**	**Not at all familiar**			**Completely familiar**
	**1**	**2**	**3**	**4**
Rehabilitation	❑	❑	❑	❑
Spleen	❑	❑	❑	❑
Medical ventilator (respirator)	❑	❑	❑	❑
Withdrawal (symptoms)	❑	❑	❑	❑

Tool 4. Knowledge of health and disease

Choose the option that you believe is the right one for each of the seven questions.

Which of the following is one of the livers main functions?

a. ❑ Detoxing of the blood.
b. ❑ Oxygenate blood
c. ❑ Urine production
d. ❑ I will ask someone else, since I'm unsure about the answer

Nephrology is the doctrine of?

a. ❑ Liver diseases
b. ❑ Kidney diseases
c. ❑ Nervous diseases
d. ❑ I will ask someone else, since I'm unsure about the answer

The last three tools concern the use of electronic devices such as computer, tablet, smartphone, smartTV or similiar.

If you use other electronic devices than a computer, then base your answers on the electronic device that you use the most in everyday life.

Tool 5. Technology familiarity

Rate on a scale from not at all familiar to completely familiar.

**How familiar are you with the following items:**	**Not at all familiar**			**Completely familiar**
	**1**	**2**	**3**	**4**
Keyboard	❑	❑	❑	❑
Settings	❑	❑	❑	❑
Operating system (e.g. Windows)	❑	❑	❑	❑
User name	❑	❑	❑	❑

Tool 6. Technology confidence

On a scale from not at all confident to completely confident, rate your use of computers.

**How confident do you feel …?**	**Not at all confident**			**Completely confident**
	**1**	**2**	**3**	**4**
Using a computer in general?	❑	❑	❑	❑
Using touchscreen?	❑	❑	❑	❑
Finding information online?	❑	❑	❑	❑

Tool 7. Incentives for engaging with technology

On a scale from completely disagree to completely agree, rate your experience of computers.

**How much do you agree or disagree with the following statements:**	**Completely disagree**			**Completely agree**
	**1**	**2**	**3**	**4**
I'm interested in using computers.	❑	❑	❑	❑
I'm fond of my computer.	❑	❑	❑	❑
I'm not afraid to try out new functions on computers.	❑	❑	❑	❑
